# Lymphocyte-to-monocyte ratio is associated with all‑cause and cardiovascular mortality among individuals with diabetes mellitus in the National Health and Nutrition Examination Survey 2003–2018 cohort

**DOI:** 10.1186/s12872-025-05088-7

**Published:** 2025-10-01

**Authors:** Anmin Ren, Shanshan Cao, Donghuo Gong, Xinkai Qu

**Affiliations:** 1 Department of Cardiology, Huadong Hospital, Fudan University, No. 221 Yan’an West Road, Shanghai, 200040 China; 2 Department of Gerontology, Huadong Hospital, Fudan University, No. 221 Yan’an West Road, Shanghai, 200040 China; 3 Shanghai Key Laboratory of Clinical Geriatric Medicine, Huadong Hospital, Fudan University, No. 221 Yan’an West Road, Shanghai, 200040 China; 4 Department of Neurology, Shanghai Pudong New Area Gongli Hospital, Shanghai, China

**Keywords:** LMR, All‑cause mortality, Cardiovascular mortality, Diabetes mellitus

## Abstract

**Background:**

Limited research has explored the association between the lymphocyte-to-monocyte ratio (LMR) and mortality in patients with diabetes mellitus. We investigated the association of the LMR with both all-cause and cardiovascular mortality in individuals with diabetes mellitus.

**Methods:**

This study enrolled participants from the 2003–2018 National Health and Nutrition Examination Survey (NHANES) cycles. Mortality data were extracted from the National Death Index records. Maximally Selected Rank Statistics (MSRS) was used to identify the best LMR cutoff that was significantly associated with the survival outcomes. Multivariate Cox regression and subgroup analyses were performed to investigate the correlations of the LMR with all-cause and cardiovascular mortality. Restricted cubic splines (RCS) analysis was used to depict the non-linear relationships of the LMR with all-cause and cardiovascular mortality. Time-dependent receiver operating characteristic (ROC) curve analysis was performed to assess the accuracy of the LMR for forecasting the survival outcomes.

**Results:**

Over the median follow-up period of 76 months, 585 of 2,327 participants died, 180 of whom died of cardiovascular mortality. The participants were divided into two groups according to the MSRS: the low LMR (≤ 2.62) and the high LMR (> 2.62) groups. The multivariate Cox regression analysis showed that the high LMR group had a significantly lower risk of all-cause mortality (hazard ratio [HR] 0.62, 95% confidence interval [CI] 0.50–0.76, *P* < 0.001) and cardiovascular mortality (HR 0.55, 95% CI 0.38–0.81, *P =* 0.003) than the low LMR group. This trend remained consistent throughout the subgroup analyses, with no significant interaction (*P*_interaction_ >0.05) observed between the LMR and these subgroup factors. The RCS regression analysis demonstrated positive non-linear relationships between the LMR and all-cause and cardiovascular mortality (both *P*_non−linear_ < 0.05) in patients with diabetes mellitus. The area under the ROC curve (AUC) for all-cause mortality was 0.858, 0.807, 0.807, and 0.802 for 1-, 3-, 5-, and 10-year survival, respectively, and the AUC for cardiovascular mortality was 0.864, 0.800, 0.815, and 0.811, respectively.

**Conclusion:**

In individuals with diabetes mellitus, high LMR correlated with a reduced risk of all-cause and cardiovascular mortality.

## Introduction

Diabetes mellitus is a Significant health concern in contemporary society. In 2021, the number of individuals diagnosed with diabetes mellitus globally had reached 529 million, resulting in a prevalence rate of 6.1% [1]. Diabetes mellitus can lead to multiorgan damage, including damage to the heart, eyes, kidneys, and extremities. This can lead to a range of complications, including coronary artery disease, diabetic ophthalmopathy, diabetic nephropathy, and limb disability [2]. Diabetes mellitus is positively associated with all-cause and cardiovascular mortality [3]. Therefore, investigating the risk factors for diabetes mellitus is essential for reducing the mortality rate among individuals with this condition.

Recent studies have indicated that inflammatory markers predict the incidence and mortality associated with cardiovascular disease [4]. The lymphocyte-to-monocyte ratio (LMR) is significantly associated with the incidence of cardiovascular disease. A previous study indicated that the LMR was significantly correlated with the severity of coronary atherosclerosis in patients with coronary artery disease, as assessed by the Gensini score [5]. Moreover, Gary et al. reported that decreased LMR was significantly associated with an increased risk of critical limb ischemia [6]. Furthermore, it was revealed that individuals with an LMR below the cutoff value of 3.1 exhibited a higher prevalence of diabetes mellitus than those with an elevated LMR, implying an interrelationship between the LMR and diabetes mellitus. A recent study suggested that elevated LMR was independently associated with a reduction in all-cause mortality among patients with obesity-related hypertension, irrespective of the presence of diabetes mellitus [7]. In addition, the LMR has been regarded as an independent predictor of rehospitalization and long-term major cardiovascular events in patients with ST-segment elevation myocardial infarction following primary percutaneous coronary intervention [8]. However, the association between the LMR and the risk of all-cause and cardiovascular mortality in patients with diabetes mellitus remains unclear. We conducted this study to evaluate the relationship of the LMR with all-cause and cardiovascular mortality in a representative dataset of patients with diabetes mellitus.

## Methods

### Study population

The National Health and Nutrition Examination Survey (NHANES) is a comprehensive cross-sectional study conducted by the National Center for Health Statistics (NCHS). The NHANES was designed to assess the health and nutritional status of a representative sample of the United States population [9]. The original NHANES was approved by the Institutional Review Board of the NCHS. All participants included in the NHANES completed the questionnaire and provided written informed consent.

**Fig. 1:**
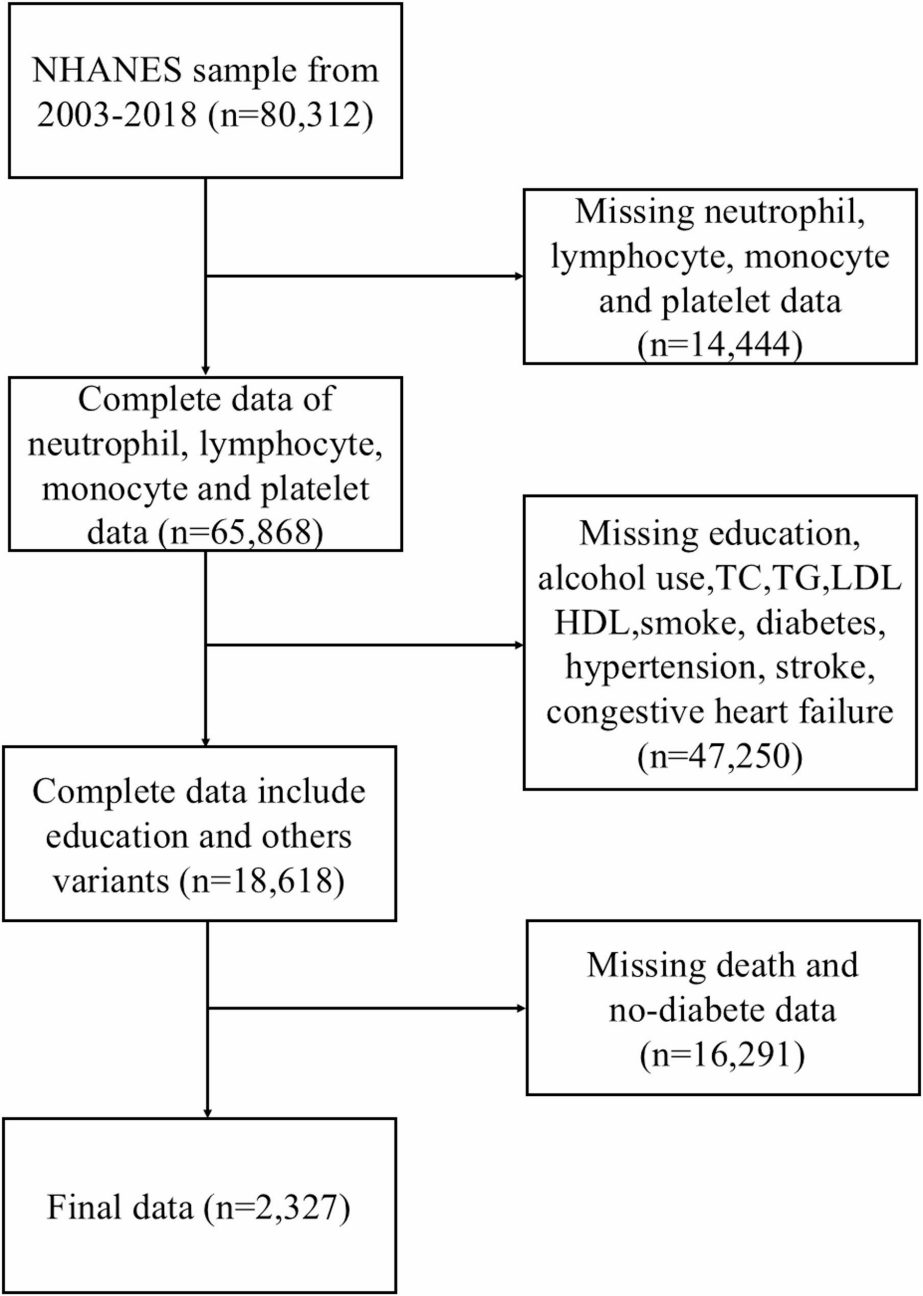
Flow chart of study participants. NHANES, National Health and Nutrition Examination Survey

The data used in the present study were derived from eight 2-year NHANES cycles conducted between 2003 and 2018, which included 80,312 participants. After excluding 14,444 participants with missing neutrophil, lymphocyte, monocyte, and platelet data, 47,250 participants with multiple missing covariates, including level of education, alcohol use, total cholesterol (TC), triglyceride (TG), low-density lipoprotein (LDL), high-density lipoprotein (HDL), smoking status, diabetes mellitus history, hypertension history, stroke history, and congestive heart failure history, were excluded. Participants without complete survival data and without diabetes mellitus were excluded (*n* = 16,291). This study of NHANES data ultimately included 2,327 participants (Fig. 1).

### Definition of diabetes mellitus and assessment of hematological parameters

Individuals who fulfilled one or more of the following criteria were classified as having diabetes mellitus: (1) fasting plasma glucose concentration of ≥ 7.0 mmol/L or a 2-hour oral glucose tolerance test result of ≥ 11.1 mmol/L; (2) random blood glucose concentration of ≥ 11.1 mmol/L; (3) glycated hemoglobin (HbA1c) of ≥ 6.5%; (4) active use of antidiabetic medication or insulin; and (5) self-reported physician diagnosis of diabetes mellitus [10]. The complete blood count is a standard laboratory test used to assess the overall health of participants and identify a broad spectrum of disorders. The methodologies used to obtain the complete blood count were based on Beckman Coulter technology. The LMR was calculated by dividing the lymphocyte count by the monocyte count.

### Mortality outcomes among the study cohort

Mortality data were obtained from the National Death Index (NDI) database of the Centers for Disease Control and Prevention (https://www.cdc.gov/nchs/data-linkage/mortality-public.htm*).* The follow-up period for each individual ranged from the participation date to the date of death or to the latest update in the NDI database on December 31, 2019. International Classification of Diseases, version 10, codes were used to identify participants with cardiovascular mortality (I00–I09, I11, I13, and I20–I51) [11].

### Covariate definitions

This study identified several covariates that may potentially influence the survival outcomes. Age, gender, race, body mass index (BMI), smoked at least 100 cigarettes in their lifetime, alcohol use, hypertension, congestive heart failure, and stroke were extracted from the demographic and health questionnaires of the NHANES. Race was categorized as Mexican–American, non-Hispanic White, non-Hispanic Black, and other race. BMI was calculated as weight in kilograms divided by height in meters squared and categorized into three groups: <25 kg/m², 25 kg/m^2^ ≤ BMI < 30 kg/m², and > 30 kg/m² [12]. Smoking status was categorized by < 100 cigarettes or ≥ 100 cigarettes in their lifetime [13]. Alcohol use was classified as having > 12 drinks per year, more than 12 times is defined as active alcohol use [14]. Hypertension, congestive heart failure, and stroke history were determined by participant self-reporting through health questionnaires [15]. Level of education was categorized as college level or higher (> high school), high school or equivalent, and below high school (< high school) [13]. Heart rate, systolic blood pressure (mmHg), diastolic blood pressure (mmHg), HbA1c (%), blood cell count (1000 cells/µL), urea nitrogen (mg/dL), serum creatinine (Scr; µmol/L), HDL, LDL, TC, and TG were all acquired from the laboratory test results.

### Statistical analysis

The statistical analysis conducted in this study utilized weighted NHANES data and accounted for a complex multi-period design [16]. The sampling weight was determined as follows: WTSAF2YR from blood lipid data ÷ 8. Continuous variables are expressed as the median with interquartile range, while categorical variables are presented as counts and percentages. Normally distributed continuous variables were compared using the independent-samples t-test, while non-normally distributed continuous variables were compared using the Mann–Whitney U test. Categorical variables were compared using the chi-square test.

The optimal LMR cutoff with the most significant correlation with the survival outcomes was determined using the Maximum Selected Rank Statistics (MSRS) from the “maxstat” package of R [10]. Restricted cubic splines (RCS) analysis with three knots was used to visualize the potential non-linearity between the LMR and all-cause and cardiovascular mortality in patients with diabetes mellitus. The relationship of the LMR with all-cause mortality and cardiovascular mortality in patients with diabetes mellitus was evaluated using the survey-weighted Cox regression analysis. Multivariate Cox regression was performed using three different models: Model 1 (unadjusted), Model 2 (adjusted for age, sex, race, BMI, smoking history, alcohol use, and level of education), and Model 3 (adjusted for age, sex, race, BMI, smoking history, alcohol use, level of education, heart rate, HbA1c, HDL, LDL, TC, TG, urea nitrogen, Scr, hypertension, and congestive heart failure). The three models were used to explore the possible confounding factors. The survival outcome probabilities were estimated using the Kaplan–Meier method and compared using the log-rank test. The study population was stratified into subgroups based on age, sex, smoking history, alcohol use, BMI, and hypertension to analyze the relationship between the LMR and mortality, while also exploring potential interaction effects. Time-dependent receiver operating characteristic (ROC) curve analysis was performed using the “timeROC” package of R to assess the accuracy of the LMR in predicting the survival outcomes at various time points [17]. Statistical analyses were performed using R (version 4.3.0) in conjunction with Zstats version 1.0 (www.zstats.net). *P* < 0.05 was considered statistically significant for all analyses.

## Results

### Baseline characteristics of the participants

**Fig. 2:**
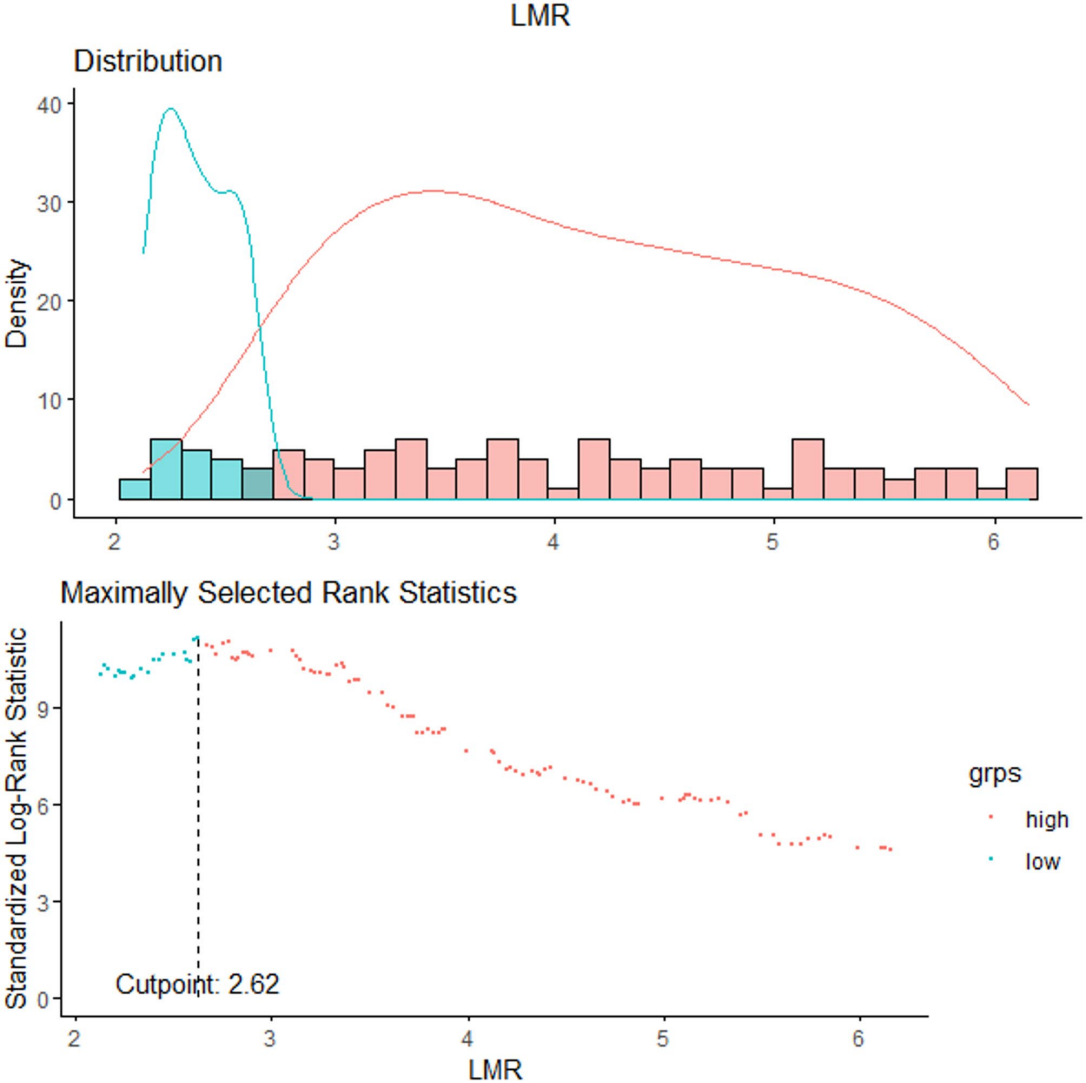
The cutoff point was determined by the maximally selected rank statistics derived from the ‘maxstat’ package

This cross-sectional study included 2,327 participants with diabetes mellitus from the 2003–2018 NHANES cycles. The participants were classified into the high LMR (> 2.62, *n* = 1,846) and low LMR (≤ 2.62, *n* = 481) groups using the optimal LMR cutoff value of 2.62, which was determined based on MSRS corresponding to the most significant association with the survival outcomes (Fig. 2). Table 1 presents the comparisons of various variables between the two groups. Compared with the low LMR group, participants in the high LMR group were younger, more frequently female, and less likely to be smokers, and they had slightly elevated heart rate and higher HbA1c, white blood cell count, lymphocyte count, platelet count, LDL, TC, and TG. Meanwhile, the neutrophil count, monocyte count, urea nitrogen, and Scr, as well as the incidence of congestive heart failure and stroke, were significantly lower in the high LMR group.

**Table 1:** Baseline characteristics of the study population

	Over all	Lower LMR	Higher LMR	*P* value
	n=2327	n=481	n=1846	
Age, y	61.00 (51.00, 70.00)	67.00 (60.00, 76.00)	58.57 (49.00, 68.00)	<0.001
Gender (%)				<0.001
Male	1190 (50.3)	337 (67.9)	853 (45.5)	
Female	1137 (49.7)	144 (32.1)	993 (54.5)	
Race (%)				<0.001
Mexican American	441 (9.3)	55 (5.1)	386 (10.5)	
Non-Hispanic White	586 (15.1)	82 (8.9)	504 (16.8)	
Non-Hispanic Black	847 (61.5)	267 (74.5)	580 (57.9)	
Other race	453 (14.1)	77 (11.5)	376 (14.8)	
Education (%)				0.272
<high school	843 (24.5)	147 (21.2)	696 (25.5)	
high school	512 (25.8)	118 (25.3)	394 (26.0)	
>high school	972 (49.6)	216 (53.5)	756 (48.6)	
Smoked at least 100 cigarettes (%)				0.006
YES	1150 (49.7)	275 (58.6)	875 (47.3)	
NO	1177 (50.3)	206 (41.4)	971 (52.7)	
Alcohol use (%)				0.199
Active alcohol user	1604 (71.0)	353 (74.2)	1251 (70.1)	
Non-Active alcohol user	723 (29.0)	128 (25.8)	595 (29.9)	
BMI, kg/m2 (%)				0.185
<25	318 (12.2)	74 (13.4)	244 (11.9)	
25-<30	728 (28.3)	161 (32.5)	567 (27.2)	
>30	1281 (59.5)	246 (54.2)	1035 (60.9)	
SBP, mmHg	128.00 (118.00, 140.00)	130.00 (118.00, 142.00)	126.00 (118.00, 140.00)	0.104
DBP, mmHg	70.00 (64.00, 78.00)	70.00 (60.00, 76.00)	72.00 (64.00, 78.00)	0.004
Heart rate	72.00 (64.00, 80.00)	70.00 (64.00, 78.00)	72.00 (64.00, 80.00)	0.014
HbA1c, %	6.90 (6.20, 7.90)	6.80 (6.20, 7.50)	6.90 (6.20, 8.15)	0.006
White blood cell, 1000 cells/ul	7.10 (6.00, 8.50)	7.00 (5.80, 8.59)	7.20 (6.10, 8.50)	0.639
Lymphocyte, 1000 cells/ul	1.90 (1.60, 2.40)	1.50 (1.10, 1.70)	2.10 (1.70, 2.60)	<0.001
Neutrophils, 1000 cells/ul	4.30 (3.40, 5.40)	4.70 (3.70, 6.10)	4.20 (3.30, 5.20)	<0.001
Monocyte, 1000 cells/ul	0.60 (0.40, 0.70)	0.70 (0.60, 0.80)	0.50 (0.40, 0.60)	<0.001
Platelet, 1000 cells/ul	233.00 (192.00, 285.00)	214.00 (175.00, 259.00)	236.00 (198.00, 289.00)	<0.001
UA, mg/dL	5.60 (4.70, 6.80)	6.20 (4.90, 7.20)	5.50 (4.60, 6.60)	<0.001
Scr, μmol/L	76.91 (63.65, 92.82)	84.86 (71.60, 106.08)	74.26 (61.88, 89.28)	<0.001
LDL, mmol/L	2.48 (1.91, 3.15)	2.33 (1.73, 2.92)	2.51 (1.94, 3.19)	<0.001
TG, mmol/L	1.49 (1.03, 2.08)	1.38 (0.96, 1.96)	1.52 (1.05, 2.13)	0.041
TC, mmol/L	4.45 (3.83, 5.25)	4.32 (3.51, 5.04)	4.50 (3.93, 5.33)	<0.001
HDL, mmol/L	1.19 (1.03, 1.45)	1.19 (1.01, 1.40)	1.19 (1.03, 1.45)	0.189
Hypertension (%)				0.063
YES	1595 (67.5)	347 (72.8)	1248 (66.0)	
NO	732 (32.5)	134 (27.2)	598 (34.0)	
Congestive Heart Failure (%)				0.001
YES	251 (9.40)	88 (14.20)	163 (8.10)	
NO	2076 (90.6)	393 (85.8)	1683 (91.9)	
Stroke (%)				0.037
YES	232 (9.5)	59 (12.5)	173 (8.7)	
NO	2095 (90.5)	422 (87.5)	1673 (91.3)	

Data are presented as number (%) or median (interquartile range)

*BMI *Body mass index, *SBP* Systolic blood pressure, *DBP* Diastolic blood pressure, *HbA1c* Glycosylated hemoglobin,* UA* Urea nitrogen, *Scr* Serum creatinine, *LMR* Lymphocyte-to monocyte ratio, *TC* Total cholesterol, *TG* Triglyceride, *LDL* Low density lipoprotein,* HDL* High density lipoprotein

### Association of the LMR with all‑cause mortality

**Fig. 3:**
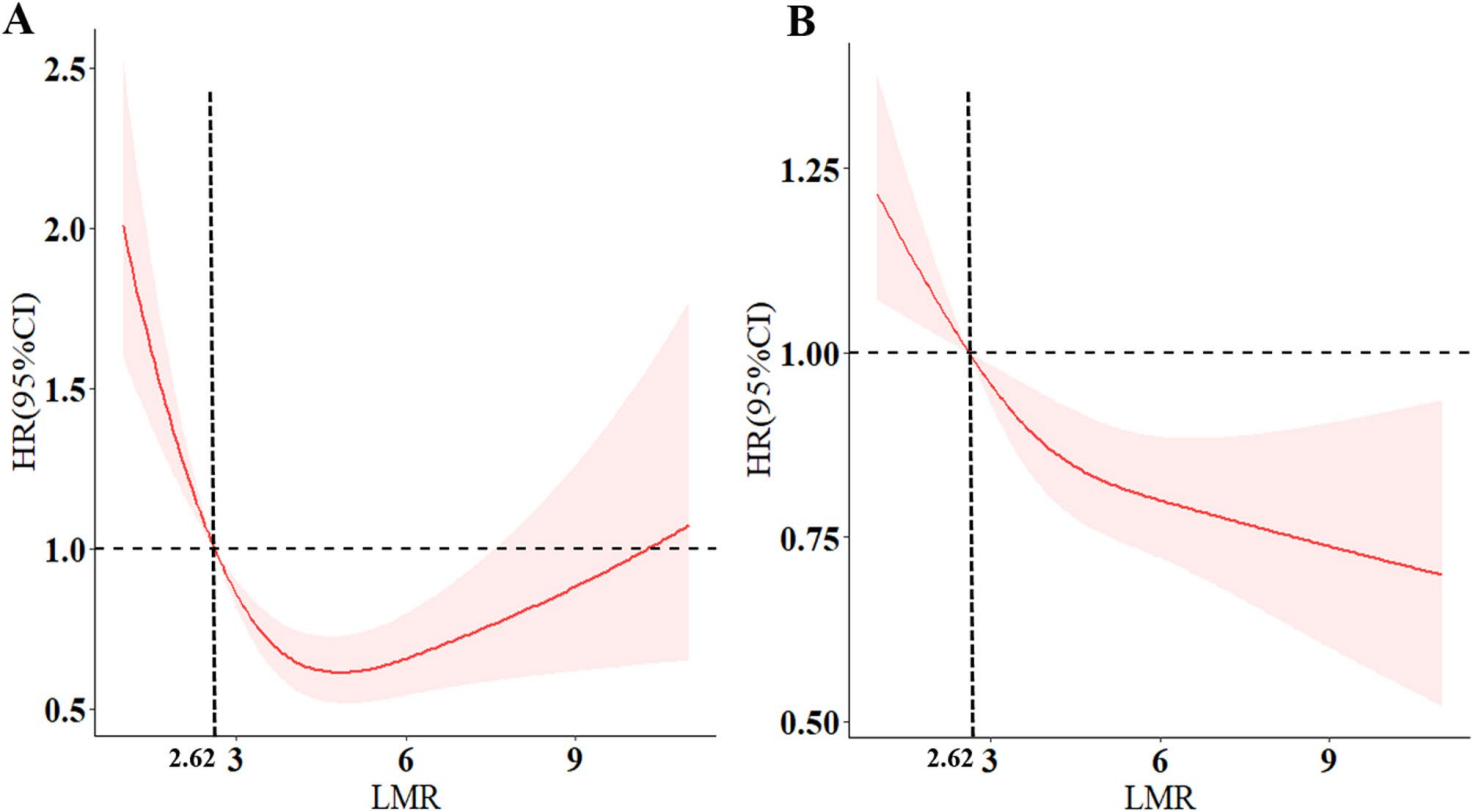
The analysis of restricted cubic spline regression (RCS) The association of LMR with all-cause (**A**) and cardiovascular mortality (**B**) among patients with diabetes. LMR: lymphocyte-to monocyte ratio; A and B adjusted for age, gender, race, BMI, smoke, alcohol use, education level, HR, HbA1c, HDL, LDL, TC, TG, UA, Scr, hypertension and congestive heart failure; both nonlinear *p* < 0.05

**Fig. 4:**
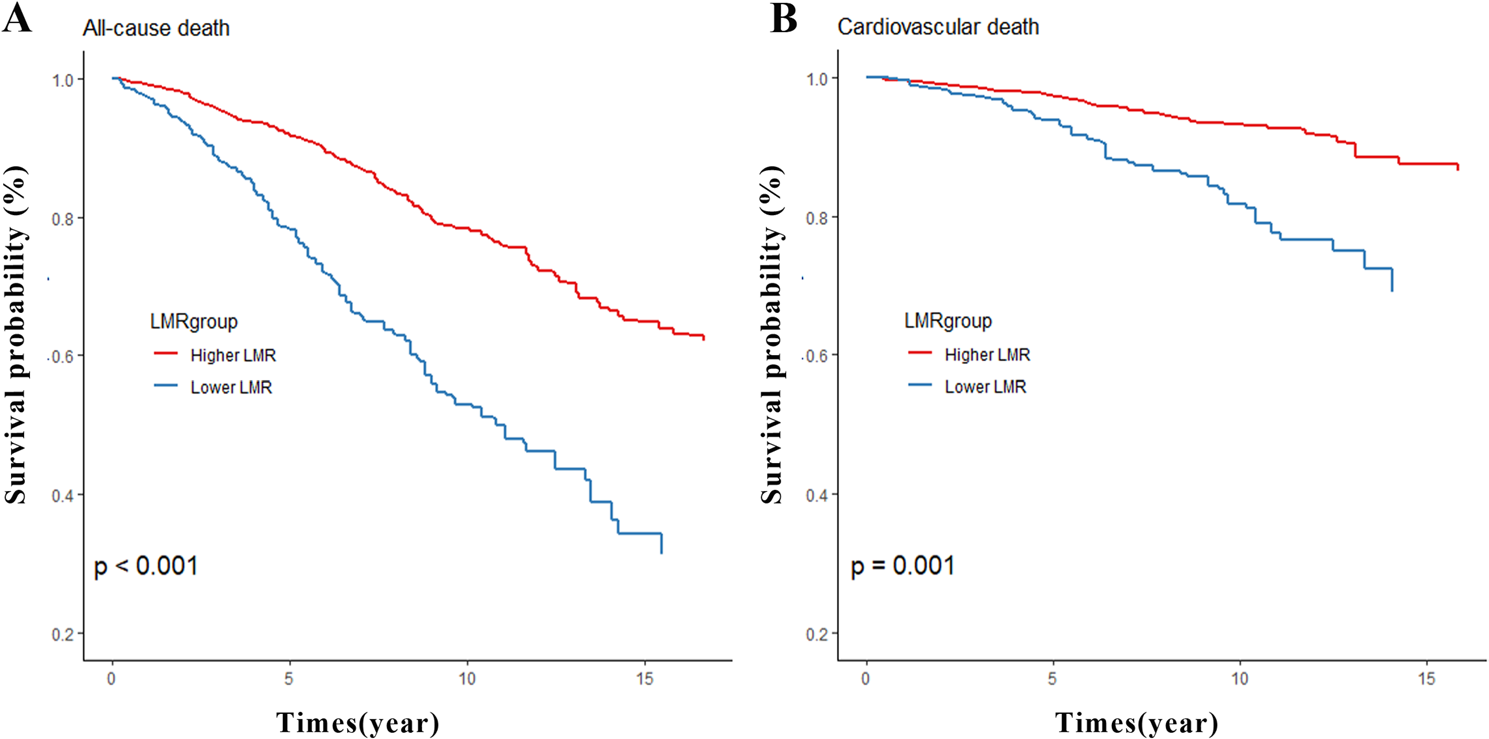
The analysis of Kaplan–Meier curves The survival rate of LMR with all-cause (**A**) and cardiovascular mortality (**B**) among patients with diabetes showed by Kaplan–Meier curves;

During the median follow-up period of 76 months, 585 of 2,327 participants died, 180 of whom died from cardiovascular mortality. The multivariate Cox regression analysis was conducted to explore the association between the LMR and all‑cause mortality. In Model 1 (unadjusted), the LMR exhibited a negative correlation with all‑cause mortality (hazard ratio [HR] 0.76, 95% confidence interval [CI] 0.71–0.80, *P <* 0.001). After LMR stratification into the low and high LMR groups, all-cause mortality was significantly lower in the high LMR group than in the low LMR group (HR 0.36, 95% CI 0.30–0.43, *P <* 0.001) (Table 2). In Model 2 (adjusted for age, sex, race, BMI, smoking history, alcohol use, and level of education), the LMR exhibited a significant negative correlation with all-cause mortality (HR 0.87, 95% CI 0.79–0.97, *P =* 0.012), and the trend observed in the high LMR group was consistent with that observed in Model 1 (HR 0.59, 95% CI 0.50–0.71, *P <* 0.001). In Model 3 (adjusted for the same variables as Model 2, plus heart rate, HbA1c, HDL, LDL, TC, TG, urea nitrogen, Scr, hypertension, and congestive heart failure), we also observed significantly lower all-cause mortality in the high LMR group than in the low LMR group (HR 0.64, 95% CI 0.50–0.82, *P* < 0.001) (Table 2). The RCS analysis revealed a significant non-linear association between the LMR and all-cause mortality in patients with diabetes mellitus (*P*_non−linear_ < 0.05) (Fig. 3A). The Kaplan‒Meier survival analysis indicated that all-cause mortality was significantly lower in the high LMR group than in the low LMR group (*P* < 0.001) (Fig. 4A). The subgroup analysis stratified by age, sex, smoking history, BMI, alcohol use, and hypertension also demonstrated a negative correlation between the LMR and all-cause mortality (Table 3). No significant interaction was observed between these specified covariates and the LMR (all *P*_interaction_ >0.05) (Table 3).

**Table 2:** The relationships between LMR and mortality in diabetes

Characteristic		Model 1	*P* value	Model 2	*P *value	Model 3	*P *value
		HR (95%CI)		HR (95%CI)		HR (95%CI)	
All-caues mortality							
LMR continuous		0.76 (0.71-0.80)	<0.001	0.87 (0.79-0.97)	0.012	0.91 (0.82-1.01)	0.087
LMR category	Lower LMR	ref		ref		ref	
	Higher LMR	0.36 (0.30-0.43)	<0.001	0.59 (0.50-0.71)	<0.001	0.64 (0.50-0.82)	<0.001
Cardiovascular mortality							
LMR continuous		0.76 (0.68-0.84)	<0.001	0.94 (0.80-1.11)	0.463	0.97 (0.76-1.23)	0.788
LMRcategory	Lower LMR	ref		ref		ref	
	Higher LMR	0.35 (0.26-0.47)	<0.001	0.56 (0.40-0.77)	<0.001	0.58 (0.36-0.93)	0.025

Model 1: unadjusted; Model 2: adjusted for age, gender, race, BMI, smoke, alcohol use and education level; Model 3: adjusted for age, gender, race, BMI, smoke, alcohol use, education level, Heart rate, HbA1c, HDL, LDL, TC, TG, UA, Scr, hypertension, congestive heart failure

*LMR* Lymphocyte-to monocyte ratio

**Table 3:** Subgroup analysis of the associations between LMR with all-cause mortality and cardiovascular mortality among diabetes

Characteristic	All-cause mortality HR(95%CI)				Cardiovascular mortality HR(95%CI)			
	Lower LMR	Higher LMR	*P* value	*P* interaction	Lower LMR	Higher LMR	*P* value	*P* interaction
Age				0.583				0.122
<60	Ref	0.44(0.21-0.93)	0.032		Ref	1.2(0.21-6.76)	0.839	
>=60	Ref	0.51(0.39-0.66)	<0.001		Ref	0.39(0.23-0.65)	<0.001	
Gender				0.428				0.963
Male	Ref	0.52(0.37-0.74)	<0.001		Ref	0.47(0.26-0.84)	0.012	
Female	Ref	0.43(0.26-0.69)	0.001		Ref	0.41(0.19-0.90)	0.026	
Smoke				0.371				0.299
YES	Ref	0.46(0.34-0.62)	<0.001		Ref	0.49(0.24-0.99)	0.001	
NO	Ref	0.48(0.31-0.77)	0.002		Ref	0.39(0.22-0.70)	0.047	
BMI				0.689				0.121
<25	Ref	0.53(0.32-0.89)	0.017		Ref	0.73(0.24-2.19)	0.574	
25-<30	Ref	0.42(0.27-0.65)	<0.001		Ref	0.35(0.14-0.89)	0.027	
>=30	Ref	0.42(0.29-0.60)	<0.001		Ref	0.35(0.19-0.65)	0.001	
Alcohol use				0.193				0.038
YES	Ref	0.41(0.30-0.55)	<0.001		Ref	0.32(0.17-0.59)	<0.001	
NO	Ref	0.53(0.33-0.86)	0.01		Ref	0.72(0.29-1.77)	0.473	
Hypertension				0.996				0.569
YES	Ref	0.43(0.32-0.59)	<0.001		Ref	0.4(0.23-0.73)	0.002	
NO	Ref	0.5(0.29-0.86)	0.012		Ref	0.45(0.17-1.21)	0.113	

HRs were adjusted for race, education level, Heart rate, HbA1c, HDL, LDL, TC, TG, UA, Scr, congestive heart failure

### Association of the LMR with cardiovascular mortality

The Cox regression analysis further determined the association between the LMR and cardiovascular mortality. In Model 1 (unadjusted), the LMR exhibited a negative correlation with cardiovascular mortality (HR 0.76, 95% CI 0.68–0.84, *P <* 0.001). Meanwhile, cardiovascular mortality was significantly lower in the high LMR group than in the low LMR group (HR 0.35, 95% CI 0.26–0.47, *P <* 0.001) (Table 2). Following adjustment (Model 2 and Model 3), the association between both LMR groups and cardiovascular mortality remained significant. The RCS analysis showed a significant non-linear association between the LMR and cardiovascular mortality in patients with diabetes mellitus (*P*_non−linear_ < 0.05) (Fig. 3B). The Kaplan‒Meier survival analysis also indicated that cardiovascular mortality was significantly lower in the high LMR group than in the low LMR group (*P* = 0.001) (Fig. 4B).

For participants aged > 60 years, subgroup analyses based on age, sex, smoking history, alcohol use, and hypertension revealed a similar association between the LMR and cardiovascular mortality. There were no significant interactions between the specified covariates and the LMR (all *P*_interaction_ >0.05) (Table 3).

### ROC analysis of the predictive value of the LMR for all-cause and cardiovascular mortality in patients with diabetes mellitus

**Fig. 5:**
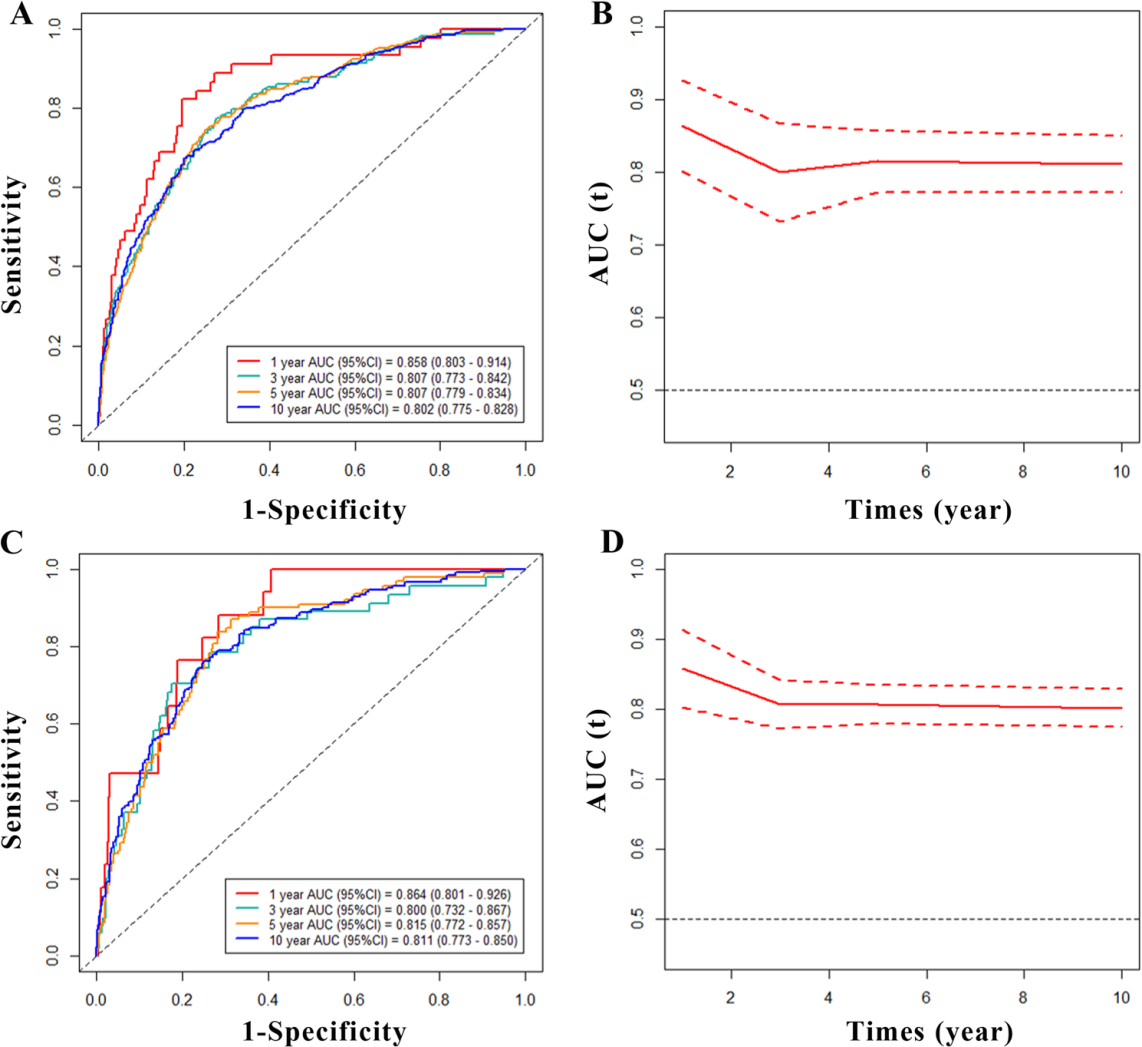
Time-dependent ROC curves and time-dependent AUC values (with 95% confidence band) of the LMR for predicting all-cause mortality (**A**,** B**) and cardiovascular mortality (**C**,** D**)

We used the time-dependent ROC curve analysis to evaluate the prognostic value of the LMR for predicting all-cause and cardiovascular mortality in individuals with diabetes mellitus. The area under the ROC curve (AUC) for the LMR was 0.858 (95% CI 0.803–0.914) for 1-year, 0.807 (95% CI 0.773–0.842) for 3-year, 0.807 (95% CI 0.779–0.834) for 5-year, and 0.802 (95% CI 0.775–0.828) for 10-year all-cause mortality (Fig. 5A and B). The AUC of the LMR was 0.864 (95% CI 0.801–0.926) for 1-year, 0.800 (95% CI 0.732–0.867) for 3-year, 0.815 (95% CI 0.772–0.857) for 5-year, and 0.811 (95% CI 0.773–0.850) for 10-year cardiovascular mortality (Fig. 5C and D).

## Discussion

This study included a total of 2,327 participants with diabetes mellitus from the 2003–2018 NHANES cycles. We observed significant negative correlations between the LMR and both all-cause and cardiovascular mortality, and LMR was an independent risk factor for both. These negative correlations remained significant after adjusting for multiple covariates. Moreover, the LMR demonstrated strong predictive value for both all-cause and cardiovascular mortality in the ROC curve analysis.

The LMR is composed of the ratio of lymphocytes to monocytes, serves as an inflammatory marker and plays a Significant role in the pathogenesis of cardiovascular diseases. T lymphocyte 1 (Th1) secrete a variety of cytokines, including IFN-γ and VCAM-1, which promote inflammatory responses and recruit leukocyte. In contrast, Th2 regulate the activation, proliferation, and myofibroblastic differentiation of stromal cells or fibroblasts, as well as matrix accumulation, ultimately contributing to scar formation in myocardial tissue [18]. Regulatory T cells (Tregs) inhibit endothelial dysfunction through the secretion of IL-10 and TGF-β, suppress the expression of pro-inflammatory factors, maintain vascular homeostasis, and secrete VEGF to promote vascular repair following injury [19]. In the early stage of atherosclerosis, monocytes are recruited to the damaged vascular endothelium by chemokines, adhere to endothelial cells, differentiate into macrophages, and ultimately develop into foam cells, contributing to the formation of vascular plaques [20]. In the development process of diabetes, these two types of cells also play a crucial role. For instance, Abdullah et al. found that T-lymphocyte depletion mitigates cardiac fibrosis in drug-induced diabetic cardiomyopathy [21]. Moreover, a novel role for circulatory factors derived from monocytes was demonstrated in the pathogenesis of cerebral microvascular lesions associated with diabetes mellitus [22]. Diabetes mellitus-induced microcirculatory impairment can further exacerbate vascular inflammation and elevate monocytes.

The monocyte-to-lymphocyte ratio has recently been identified as being associated with various diabetes-related diseases and complications, including diabetic retinopathy [23], diabetic foot ulcer [24], and diabetic nephropathy [25]. Thus, we propose that the LMR may serve as a critical biomarker in the context of diabetes mellitus-related diseases. A previous study indicated that both the lymphocyte count and LMR exerted a protective influence on the incidence and complications associated with macrovascular disease in patients with diabetes mellitus [26]. In another study, the LMR served as a predictive indicator for the necessity of surgical intervention in cases of diabetic foot infection [27]. Meanwhile, we believe that the predictive value of LMR in the diabetic population is superior to that of MLR, and this belief is supported by the following reasons: The high mortality rate of cardiovascular diseases in patients with diabetes is directly associated with chronic low-grade inflammation and immunosuppression [28]. Lymphocyte serves as the core mechanism underlying diabetic cardiovascular events and plays a dominant role in this process. Decreasing in LMR directly reflects the exacerbation of immunosuppression. Conversely, monocytes exhibit relatively low specificity and can be readily influenced by various non-cardiovascular comorbidities, potentially compromising their accuracy as an independent predictive marker. In terms of clinical prediction, this study demonstrates that LMR exhibits significant predictive effect on cardiovascular mortality in diabetic patients (Fig. 5), while few previous MLR studies did not distinguish cardiovascular mortality [25]. The present study provides novel insights into the relationship between the LMR and survival in patients with diabetes mellitus, demonstrating that the LMR may serve as an independent risk factor for all-cause mortality and cardiovascular mortality.

Atherosclerosis is associated with a range of cardiovascular mortality events, and the inflammation driven by various types of blood cell plays a crucial role in atherosclerotic plaque development [29]. In response to endothelial injury, monocytes and macrophages infiltrate the arterial walls, where they differentiate and contribute to plaque formation through the secretion of pro-inflammatory cytokines, such as tumor necrosis factor-α and interleukin-6 [30]. However, different research results exist regarding the impact of the LMR on vascular plaques. Some studies have indicated that the LMR serves as an indicator for maintaining vascular plaque stability. For instance, Wang et al. showed that the LMR was independently negatively associated with the enhancement of plaques in intracranial atherosclerotic stenosis, and an LMR of ≤ 4.0 served as a predictor of plaque instability [31]. Another study showed that a low LMR was associated with a higher coronary artery calcification score and an increased prevalence of coronary heart disease [32]. However, another study showed that the LMR was significantly elevated in individuals with diabetic coronary artery disease compared to those with non-diabetic coronary artery disease [33]. In the present study, we divided the LMR into two categories based on the survival outcomes with a cutoff of 2.62. We found that people with an LMR of < 2.62 had higher cardiovascular disease mortality (Tables 2 and 3). However, the predictive association between the LMR and cardiovascular mortality in individuals aged < 60 years was non-significant (*P* = 0.839, Table 3). The lack of experimental samples from middle-aged individuals and the fact that younger blood vessels are more resistant to inflammatory factors may explain this phenomenon.

Lipid concentrations and inflammatory cells collaboratively contribute to the pathogenesis of atherosclerotic plaque formation. Lipid deposition at the vascular wall enhances the recruitment of inflammatory cells, including leukocytes, neutrophils, and monocytes, subsequently promoting plaque growth [34]. A previous investigation into the association between the LMR and all-cause mortality in patients with hypertension revealed a significant correlation between the LMR and various lipid parameters, with an increasing trend in TC, TG, and LDL concentrations corresponding to a higher LMR across the quartiles groups [7]. This finding is noteworthy, and our study yielded comparable results. Specifically, we showed that the concentrations of TC, TG, and LDL were significantly elevated in the high LMR group compared with the low LMR group (Table 1). However, conflicting research findings have also been reported. In a cohort study examining carotid plaques, the LMR was lower in the plaque group, indicating a protective predictive role for atherosclerosis, while lipid indicators, such as TC and TG, were elevated [35]. This intricate relationship underscores the necessity for further investigation into the association between the LMR and the lipid profile.

The strengths of this study lie in its extended follow-up period and large sample size. Moreover, the uniformity of data sources within the NHANES database avoided the potential for relevant statistical error in this study. However, this study also has several limitations that should be considered. First, all of the laboratory data were limited to cross-sectional observations in the United States. In light of the necessity for further validation of the broader applicability of the research findings, subsequent investigations are essential. Second, all data regarding hypertension, heart failure, and stroke were collected through questionnaires, and their validity requires further verification.

## Conclusion

In conclusion, we conducted an analysis of 2,327 patients with diabetes mellitus from the NHANES database (2003–2018) to elucidate the association between the LMR and the risk of all-cause and cardiovascular mortality during long-term follow-up. Our findings indicate a significant correlation that is relevant to the clinical prevention and treatment of diabetes mellitus, warranting further exploration of the association between the LMR with both all-cause and cardiovascular mortality.

## Data Availability

The NHANES database is available at:” https ://www.cdc.gov/nchs/nhanes/“ about nhanes.htm. The datasets used and/or analyzed during the current study are available from the corresponding author on reasonable request.

## Abbreviations

NHANES: National Health and Nutrition Examination Surveys
CI: Confidence interval
OR: Odds ratio
NDI: National death index
BMI: Body mass index
LMR: Lymphocyte-to-monocyte ratio
MSRS: Maximally Selected Rank Statistics
RCS: Restricted cubic splines
ROC: Receiver operating characteristic
TC: Total cholesterol
TG: Triglyceride
LDL: Low-density lipoprotein
HDL: High-density lipoprotein
HbA1c: Glycated hemoglobin
UA: Uric acid
Scr: Serum creatinine
